# Molecular investigation, using chromosomal microarray and whole exome sequencing, of six patients affected by Williams Beuren syndrome and Autism Spectrum Disorder

**DOI:** 10.1186/s13023-019-1094-5

**Published:** 2019-05-31

**Authors:** Julie Masson, Caroline Demily, Nicolas Chatron, Audrey Labalme, Pierre-Antoine Rollat-Farnier, Caroline Schluth-Bolard, Brigitte Gilbert-Dussardier, Fabienne Giuliano, Renaud Touraine, Sylvie Tordjman, Alain Verloes, Giuseppe Testa, Damien Sanlaville, Patrick Edery, Gaetan Lesca, Massimiliano Rossi

**Affiliations:** 1 Service de Génétique, Centre de Référence Anomalies du Développement, Hospices Civils de Lyon, Bron, France; 2 Centre de Recherche en Neurosciences de Lyon, GENDEV Team, INSERM U1028, CNRS UMR5292, Université Claude Bernard Lyon 1, 59 boulevard Pinel, 69677 Bron cedex, France; 3 Centre de Référence GénoPsy, Service Hospitalo-Universitaire, CRMR Maladies Rares à Expression Psychiatrique, Centre Hospitalier le Vinatier, Pôle Ouest, Bron, Université Lyon 1, Lyon, France; 4 Service de génétique médicale, CHU de Poitiers, and EA 3808, Université de Poitiers, Poitiers, France; 5 Service de génétique, CHU de Nice, Nice, France; 6 Service de génétique clinique, chromosomique et moléculaire, CHU Saint-Etienne, Saint Priez en Jarez, France; 7 Pôle Hospitalo-Universitaire de Psychiatrie Enfant et Adolescent (PHUPEA), Centre Hospitalier Guillaume Régnier, Université Rennes 1, Rennes, France; 8 Laboratoire de Psychologie de la Perception (LPP), CNRS UMR 8158, Université Paris Descartes, Paris, France; 9 Département de Génétique, APHP-Robert DEBRE University Hospital, USPC University and INSERM UMR1141, Paris, France; 10 Department of Oncology and Hemato-Oncology, University of Milan, Milan, Italy; 11 European Institute of Oncology, Milan, Italy

**Keywords:** Autism Spectrum Disorder, Williams Beuren syndrome, 7q11.23 microdeletion, *GTF2I*, Whole exome sequencing

## Abstract

Williams Beuren syndrome (WBS) is a multiple malformations/intellectual disability (ID) syndrome caused by 7q11.23 microdeletion and clinically characterized by a typical neurocognitive profile including excessive talkativeness and social disinhibition, often defined as “overfriendliness” and “hyersociability”. WBS is generally considered as the polar opposite phenotype to Autism Spectrum Disorder (ASD). Surprisingly, the prevalence of ASD has been reported to be significantly higher in WBS (12%) than in general population (1%). Our study aims to investigate the molecular basis of the peculiar association of ASD and WBS. We performed chromosomal microarray analysis and whole exome sequencing in six patients presenting with WBS and ASD, in order to evaluate the possible presence of chromosomal or gene variants considered as pathogenic.

Our study shows that the presence of ASD in the recruited WBS patients is due to i) neither atypically large deletions; ii) nor the presence of pathogenic variants in genes localized in the non-deleted 7q11.23 allele which would unmask recessive conditions; iii) moreover, we did not identify a second, indisputable independent genetic diagnosis, related to pathogenic Copy Number Variations or rare pathogenic exonic variants in known ID/ASD causing genes, although several variants of unknown significance were found. Finally, imprinting effect does not appear to be the only cause of autism in WBS patients, since the deletions occurred in alleles of both maternal and paternal origin.

The social disinhibition observed in WBS does not follow common social norms and symptoms overlapping with ASD, such as restricted interests and repetitive behavior, can be observed in “typical” WBS patients: therefore, the terms “overfriendliness” and “hypersociability” appear to be a misleading oversimplification.

The etiology of ASD in WBS is likely to be heterogeneous. Further studies on large series of patients are needed to clarify the observed variability in WBS social communication, ranging from excessive talkativeness and social disinhibition to absence of verbal language and social deficit.

**Electronic supplementary material:**

The online version of this article (10.1186/s13023-019-1094-5) contains supplementary material, which is available to authorized users.

Williams Beuren syndrome (WBS) is a multiple malformations/intellectual disability (ID) syndrome clinically characterized by typical facies, vascular involvement including arterial stenosis and hypertension, and global developmental delay. The prevalence of WBS is reported to be about 1/7500 [1]. This condition is caused by a 7q11.23 microdeletion of 1.4–1.55 megabases (Mb) (about 90–95% of patients) or 1.84 Mb (about 5–10% of patients), including 26–28 genes [1].

WBS patients generally show a neurological involvement including mild to moderate ID, attention problems, difficulties in visuospatial construction, anxiety and phobias. The hallmarks of the typical WBS neurocognitive profile are excessive talkativeness and social disinhibition, often defined as “overfriendliness” and “hyersociability” [1]; hence, WBS is generally considered as the polar opposite phenotype to Autism Spectrum Disorder (ASD) [2]. Among the genes included in the WBS critical region, *GTF2I* is a strong candidate to explain the typical WBS neurocognitive profile [3, 4].

Surprisingly, some WBS patients have been reported to show severe ASD [2, 5] and, overall, the prevalence of ASD has been reported to be significantly higher in WBS (12%) than in general population (1%) [6]. Our study aims to investigate the molecular basis of the peculiar association of ASD and WBS.

We recruited 6 patients with WBS and ASD. The diagnosis of WBS was confirmed by fluorescent in situ hybridization. All patients met formal ASD criteria. The diagnosis of ASD was set by clinical psychiatric assessment according to ICD-10 (International Classification of Diseases, World Health Organization, Genève, Switzerland, 1993) and DSM-5 (American Psychiatric Association, Washington, D.C., United States, 2013) and confirmed by standard direct evaluation scales (Autism Diagnostic Observation Schedule/ADOS scale for patients 1–4 and Childhood Autism Rating Scale/CARS for patients 5 and 6) (Table 1) and parental interviews (Autism Diagnostic Interviews-ADI/ADI-R for patients 1–5). This approach, combining information from multiple sources based on a clinical assessment and the administration of the ADOS/CARS/ADI improves the confidence in the diagnosis of ASD. All patients exhibited symptoms from the social affect and restricted/repetitive behaviors domains and were considered as impaired in all dimensions of ASD, avoiding biases due to WBS characteristics rather than to ASD profile [7]. Parental samples were available for 5/6 patients. All parents were asymptomatic. Fragile X syndrome molecular analysis was negative in all patients. Families gave written informed consent and the study was approved by the ethical committee of our institution (IRB 11263).

**Table 1:** Main features of patients affected by Williams Beuren syndrome (WBS) and Autism Spectrum Disorder (ASD)

WBS patients	1	2	3	4	5	6
Gender	M	M	F	M	M	M
Typical dysmorphic features	+	+	+	+	+	+
Age at last examination	15y6m	23y9m	9y10m	18y	10y8m	16y
ASD	+	+	+	+	+	+
ADOS	Module 1	Module 1	Module 1	Module 3	–	–
Communication (cut-off autism)	5 (≥4)	4 (≥4)	6 (≥4)	3 (≥3)	–	–
Reciprocal social interaction (cut-off autism)	13 (≥7)	11 (≥7)	12 (≥7)	9 (≥6)	–	–
Communication + social interaction total (cut-off autism)	18 (≥12)	15 (≥12)	18 (≥12)	12 (≥10)	–	–
Play	4	4	4	3	–	–
Repetitive behaviors and stereotyped patterns	4	3	5	2	–	–
CARS (cut-off: mild/moderate ASD > 30; severe ASD > 37)	–	–	–	–	34	45,5
Motor stereotypies	+	+	+	+	+	+
Speech	No speech	No speech	Few words	Sentences	Sentences	Few words
Fragile X molecular analysis	N	N	N	N	N	N
7q11.23 typical deletion	+	+	+	+	+	+
Parental origin of the deleted 7q11.23 allele	Maternal	Maternal	–	Maternal	Maternal	Paternal

+: present; *N* negative. *ADOS* Autism Diagnostic Observation Schedule, *CARS* Childhood Autistic Rating Scale. -: not available

We made a few alternative hypotheses to explain the occurrence of ASD in a subset of WBS patients: i) atypically large 7q11.23 deletions including additional genes, as previously reported [8]; ii) rare pathogenic variants in genes located within the deletion, in particular *GTF2I,* on the basis of its previous specific association to ASD (PMID: 22048961) and its overriding role in the molecular impact of 7q11.23 Copy Number Variations (CNVs) [4, 5]; iii) additional pathogenic CNVs or rare intragenic pathogenic variants located in other chromosomal regions with various inheritance patterns (autosomal recessive, X-linked, de novo autosomal dominant); given the small number of patients recruited, we focused on rare exonic variants considered to be pathogenic according to the criteria of the American College of Medical Genetics and Genomics (ACMG) [9].

In order to evaluate these hypotheses, we performed chromosomal microarray analysis (CMA) in the 6 index patients and whole exome sequencing (WES) in five trios (WBS patients and their unaffected parents) and one single patient (patient 3, whose parental samples were not available). For 4 patients (patients 1, 2, 3 and 6) CMA was performed with a custom 244 K microarray (Agilent Technologies, Santa Clara, CA, USA) enriched in oligonucleotides in the critical region for the WBS (28,147 oligonucleotides were spotted in the 7q11.23 region between position: 67558714–79,837,248 pb (hg19)). For patients 4 and 5, CMA was performed with a 105 K microarray (Agilent Technologies) and a Constitutional Chip 4.0 BAC array (Perkin Elmer, Wellesley, MA, USA) respectively. CMA procedures were performed in accordance with the manufacturer’s instructions. For WES, genomic DNA was captured using Agilent in-solution enrichment methodology (SureSelect Human All Exon Kits Version 5, Agilent) with their biotinylated oligonucleotides probes library (Human All Exon v5–50 Mb, Agilent), followed by paired-end 75 bases massively parallel sequencing on Illumina HiSEQ 2500. The bioinformatic analyses were performed using Papillyon, an in-house script which follows the Broad Institute recommendations. Briefly, the reads were trimmed with Trimmomatic and aligned against the hg19 version of the human genome using BWA-MEM. The variant calling was performed using GATK HaplotypeCaller (SNV/Indels detection), and DeCovA (CNV detection). GenoFilter (an in-house script) was used to filter the identified variants according to the hypothesized possible inheritance patterns (autosomal recessive; de novo; and X-linked inheritance). Then on all exome data, we filtered variants that had a depth lower than 5X, intronic variants, variants with an occurrence > 3 in the ExAC database (the ACMG recommendation for classifying a de novo dominant variant as pathogenic is its absence from population database like ExAC, 1000 genomes, dbSNP), to keep only rare exonic variants. Moreover, we looked for possible cerebral expression (GTEx) and used pathogenic prediction based on in silico methods (Sift, Polyphen 2, and Mutation Taster) to help us to conclude about the pathogenic role, in order to select rare exonic pathogenic variants.

CMA confirmed the presence of typical 7q11.23 deletions in all patients and ruled out other additional pathogenic CNVs (> 50 kb). Patient 1 had a variant of unknown significance (VOUS) of 386 kb: arr[GRCh37] 10q26.3(134545665_134931858)× 3, containing 8 Refseq genes, among whom 3 are referenced in OMIM; none of them were of interest for ASD or other neurodevelopmental disorders. Decipher reports several individuals with duplications of this region; however, the referral clinicians classified them as likely benign and most of them were inherited from a healthy parent.

WES did not disclose any pathogenic intragenic variant either in *GTF2I* or in other genes in the non-deleted 7q11.23 allele, as well as in 450 known genes causing ID or ASD (gene list available in Additional file 1). Concerning whole exome analysis, based on Genofilter analysis, we reviewed in average for each patient with trio exome analysis: 78 variants for recessive inheritance hypothesis, 29 variants for de novo hypothesis and 11 variants for X linked inheritance hypothesis. Moreover, after filtering to look for rare exonic variant, we reviewed 9 variant, on average, for each patient with trio exome analysis, and 144 variants for the patient with solo exome analysis. We considered 2 de novo single nucleotide variants (SNV) (1 in patient 1 and 1 in patient 2) as well as 4 SNV in patient 3 (who was sequenced on a solo basis) for their expression in brain tissue or their association with neurodevelopmental disorders; according to the ACMG, these variants were considered as VOUS (Table 2). In particular, the SNV in *PDPK1* seemed to be interesting: it was predicted as pathogenic by all in silico methods, it had a cerebral expression and it was de novo. Nevertheless, to the best of our knowledge, this variant has not been reported to date; only one article reported a large deletion encompassing *PDPK1* and 2 other genes in 6 patients presenting with developmental delay, ID and seizures. Overall, this SNV is currently considered as a VOUS.

**Table 2:** Annotation of 6 exonic variants of unknown significance, found by WES in the recruited patients

WBS patients	1	2	3	3	3	3
Exome analysis	Trio	Trio	Solo	Solo	Solo	Solo
Variant	Chr16:2647248 A > G	Chr22:38642099A > G	Chr3:196612953 T > A	Chr6:15496807 G > C	Chr6:30919581 C > T	Chr13:35716473 A > G
Gene	*PDPK1*	*TMEM184B*	*SENP5*	*JARID2*	*DPCR1*	*NBEA*
Transcript	NM_002613.4	NM_001195072.1	NM_152699.4	NM_004973.3	NM_080870.3	NM_015678.4
Nucleotide variant	c.1526A > G	c.2 T > C	c.901 T > A	c.1351G > C	c.3340C > T	c.2404A > G
Protein variant	p.Lys509Arg	p.Met1?	p.Cys301Ser	p.Glu451Gln	p.Pro1114Ser	p.Thr802Ala
Variant effect	Missense	Start lost	Missense	Missense	Missense	Missense
Genotype	Heterozygous	Heterozygous	Heterozygous	Heterozygous	Heterozygous	Heterozygous
ExAC occurrence	0	0	0	0	1	0
Inheritance	de novo	de novo	ND	ND	ND	ND
Cerebral expression (GTEx)	+	+	+	+	–	+
SIFT, Polyphen2, Mutation Taster	P, P, P	C, B, P	B, B, P	C, C, P	NA, I, B	B, I, P
Arguments	Reported in neurodevelopmental syndrome [12]	Gene function [13]	Animal study [14]	Reported in association with autism [15, 16]	Reported in association with neuropsychiatric disorders [17]	Autism candidate gene [18, 19]

*WES* whole exome sequencing, *WBS* Williams Beuren Syndrome, *NA* not available, *ND* not done, *P* pathogenic, *C* contradictory, *B* benign, *I* intermediary

DeCova did not allow us to detect any CNV because of its poor sensitivity due to WES depth heterogeneity.

The study of single-nucleotide polymorphisms in the five trios, showed that the deletion was of maternal origin in 4/5 cases.

In conclusion, our study shows that the presence of ASD in the recruited WBS patients is due to i) neither atypically large deletions; ii) nor the presence of pathogenic variants in genes localized in the non-deleted allele which would unmask recessive conditions; iii) moreover, we did not identify a second, indisputable independent genetic diagnosis, related to pathogenic CNVs or rare pathogenic exonic variants in known ID/ASD causing genes, although several variants of unknown significance were found. Finally, these results did not suggest an imprinting effect as the only cause of autism in patients with WBS, since the deletion occurred in alleles of both maternal and paternal origin.

The WBS neuropsychological profile is characterized by social disinhibition and excessive empathy which do not follow common social norms; therefore, the terms “overfriendliness” and “hypersociability” appear to be a misleading oversimplification. Excessive talkativeness is often not directed toward others, and verbal communication can be therefore impaired in WBS as in ASD. Furthermore, WBS patients can show other symptoms overlapping with ASD, such as restricted interests, repetitive behavior and hyperacousia, even in the absence of a formal diagnosis of ASD [1, 7, 10].

Our study had some limitations. Firstly, we could not exclude some small differences in the deletion breakpoints due to the presence of repeated duplicated flanking regions that are not easily mapped and sequenced. In other words, we could not rule out the hypothesis that *GTF2I* or its regulatory regions may be disrupted in WBS patients with ASD and not in those presenting with a classical neuropsychological profile. Secondly, given the small number of patients recruited, we focused on rare exonic variants considered to be pathogenic and we could not evaluate the possible role of second hits currently not classified as pathogenic, frequent variants or predisposition factors. Thirdly, we could not exclude that the etiology of ASD in WBS is heterogeneous. In this respect, the hypothesis that an imprinting effect might influence the occurrence of autistic features in a subset of WBS patients cannot be excluded.

Nevertheless, it is interesting to notice that Kopp et al. recently reported the characterization of 85 WBS patients by WES: even if the recruited patients did not undergo a formal evaluation for ASD, the authors did not find any significant variant contributing to the WBS social phenotype [11], this fitting with the conclusions of the present study.

We hope that further molecular studies by whole genome sequencing, functional expression studies on Induced Pluripotent Stem Cells [3], or studies on large series of patients exploring complex mechanisms including multi-gene inheritance, RNA modification or gene-environment interactions, will help to explain the observed variability in WBS social communication, ranging from excessive talkativeness and social disinhibition to absence of verbal language and social deficit.

## Additional file

Additional file 1: List of ID/ASD genes. (XLSX 23 kb)

## Abbreviations

ADI-R: Autism Diagnostic Interview-Revised scale
ADOS: Autism Diagnostic Observation Schedule scale
ASD: Autism Spectrum Disorder
CARS: Childhood Autism Rating Scale
CMA: Chromosomal Microarray Analysis
ICD: International Classification of Diseases
ID: Intellectual disability
VOUS: Variant Of Unknown Significance
WBS: Williams Beuren syndrome
WES: Whole Exome Sequencing
